# TECAR Therapy Associated with High-Intensity Laser Therapy (Hilt) and Manual Therapy in the Treatment of Muscle Disorders: A Literature Review on the Theorised Effects Supporting Their Use

**DOI:** 10.3390/jcm11206149

**Published:** 2022-10-19

**Authors:** Dan Alexandru Szabo, Nicolae Neagu, Silvia Teodorescu, Corina Predescu, Ioan Sabin Sopa, Loredana Panait

**Affiliations:** 1 Department of Human Movement Sciences, George Emil Palade University of Medicine, Pharmacy, Science, and Technology of Targu Mures, 540139 Targu Mures, Romania; 2 Department ME1, Faculty of Medicine in English, George Emil Palade University of Medicine, Pharmacy, Science, and Technology of Targu Mures, 540139 Targu Mures, Romania; 3 Department of Doctoral Studies, National University of Physical Education and Sports, 060057 Bucharest, Romania; 4 Department of Special Motor and Rehabilitation Medicine, National University of Physical Education and Sports, 060057 Bucharest, Romania; 5 Department of Environmental Sciences, Physics, Physical Education and Sports, “Lucian Blaga” University Sibiu, 550012 Sibiu, Romania

**Keywords:** TECAR therapy, high-intensity laser therapy, manual therapy, muscle disorders

## Abstract

Background: It has been estimated that between 30 and 50 per cent of all injuries that take place throughout participation in a sport are the consequence of soft tissue injuries, and muscle injuries are the primary cause of physical disability. Methods: The current literature review was designed between October 2021 and April 2022, according to the PRISMA standards, using the PubMed, Scopus, and Web of Science databases. At the screening stage, we eliminated articles that did not fit into the themes developed in all subchapters of the study (*n* = 70), articles that dealt exclusively with orthopaedics (*n* = 34), 29 articles because the articles had only the abstract visible, and 17 articles that dealt exclusively with other techniques for the treatment of musculoskeletal disorders. The initial search revealed 343 titles in the databases, from which 56 duplicate articles were automatically removed, and 2 were added from other sources. Results: The combination of these three techniques results in the following advantages: It increases joint mobility, especially in stiff joints, it increases the range of motion, accelerates tissue repair, improves tissue stability, and extensibility, and it reduces soft tissue inflammation (manual therapy). In addition, it decreases the concentration of pro-inflammatory mediators and improves capillary permeability, resulting in the total eradication of inflammation (HILT). It warms the deep tissues, stimulates vascularity, promotes the repose of tissues (particularly muscle tissue), and stimulates drainage (TECAR). Conclusions: TECAR therapy, combined with manual therapy and High-Intensity Laser therapy in treating muscle diseases, presented optimal collaboration in the recovery process of all muscle diseases.

## 1. Introduction

According to the World Health Organization, rehabilitation can be defined as a set of interventions designed to optimise body functions and reduce the disabilities of people with various health problems [1]. Due to the awareness of the importance of functional rehabilitation and medical recovery, the availability of rehabilitation services is constantly increasing along with increases in chronic diseases and disabilities [2]. Medical recovery aims to increase the quality of life, ensuring, in addition to gaining functional independence, the reintegration of the individual into society, with this being one of the main objectives set at the beginning of the recovery program [3,4,5].

Muscle injuries are the most common cause of physical disability, especially in sports. It was calculated that 30 to 50% of all injuries that occur during the practice of a sport are the result of soft tissue injuries [6].

Although non-surgical athletes with muscle injuries have a fair prognosis after therapy [7], the severe implications of treatment failure can be dramatic for the athlete, delaying the resumption of physical exercise by weeks or even months [8].

Skeletal muscles represent about 40% of the cumulative load of the human body [9]. Its composition consists of several individual fibres grouped in a muscle spindle; this gives the skeletal muscle a striated appearance. A single muscle fibre comprises actin and myosin fibres covered by a cell membrane (sarcolemma). These fibres are the functional unit of the organ, leading to contraction and relaxation. There are two significant classifications of skeletal muscles: type I (slow oxidative) and type II (rapid contraction) [10]. The great diversity is based on their purpose; the structure of skeletal muscles causes differences in the pace and duration of contractions in distinct muscle groups [8]. Skeletal muscles endorse the skeletal system to maintain posture and control voluntary movement. Skeletal muscles also contribute to energy metabolism and storage [11]. Some muscles cross one or more joints to generate movement. Muscles with tonic or postural function are generally wide, flat, and localised muscles in a single joint, with a low rate of contraction and an ability to generate and maintain a high contractile force. They are generally located in deeper compartments [8].

Muscles crossing two joints have a higher speed of contraction and a more remarkable ability to change their length but a lower ability to withstand tension. They are generally located in superficial compartments [7].

The current classification of muscle lesions divides them into slight, mild, and severe, depending on the medical characteristics showcased [12].

Mild muscle injuries (grade I) affect only some muscle fibres, with slight oedema and embarrassment, escorted by a small or no lack of the ability or restriction of motion during muscular contraction; it is impossible to manipulate any muscle deficiency. Although the discomfort does not trigger a significant functional disability, the athlete is not recommended to continue activities due to the high risk of the magnitude of the trauma increasing.

Moderate muscle damage (grade II) causes more considerable muscle damage, with a noticeable function loss (ability to contract). A small muscular defect or gap can be palpated at the site of damage, and a minor nearby hematoma with potential bruising forms within two to four days. The progression of healing usually lasts two to three weeks, and after about a month, the convalescent might gradually resume physical exercise [11].

Injuries that extend severe injuries occur over the whole cross-section of the muscle, resulting in the total loss of muscular function and acute discomfort, regarding muscle injuries (grade III) and muscle tears, respectively. An apparent deficiency of muscle structure and bruising is usually extensive and placed at a distance from the site of the injury. The time required to heal such lesions varies between four and six weeks. This injury requires intensive rehabilitation for extended intervals (up to three or four months). Such people may experience discomfort for months following the procedure onset and treatment of the lesion.

Muscle cramps lead to continuous, involuntary, painful, and localised contractions of a whole muscle faction, a single muscle, or selected muscle fibres. In healthy persons or the presence of diseases, cramps might endure from seconds to a minute for idiopathic or recognised reasons. The palpation of the cramped muscle region reveals a knot [12].

Early mobilisation induces an increase in local vascularity in the lesion area, better regeneration of muscle fibres, and better parallel orientation of regenerated myofibrils concerning movement restrictions [13]. However, re-injury at the initial site of trauma is common if active mobilisation is started immediately after injury [14].

Muscle fatigue is one of the main factors in decreasing muscle flexibility [15]. Numerous ways have been used over time to facilitate muscle recovery from fatigue, such as stretching techniques [16], massages, active recovery [17], contrast water therapy [18], cryotherapy [19], and thermotherapy [20].

This review aims to highlight the multitude of physiotherapy procedures in treating muscle diseases in athletes and non-athletes. Due to the produced effects, manual therapy combined with HILT or TECAR therapy, or even all three therapies used in a treatment plan, can provide optimal muscle recovery in a shorter time; therefore, the use of combined and not isolated recovery procedures is a better alternative in muscle recovery.

Moreover, we aim to reintroduce manual therapy, a relatively old technique used in physical therapy, and to use it as an adjunct technique in the therapy of muscle conditions, along with new techniques used in physical therapy, such as HILT and especially TECAR therapy. Therefore, another purpose of this review is to combine the old techniques of physical therapy with the new ones to find a treatment plan that is as effective as possible in treating muscle diseases.

The novelty of this review consists of the association of these recovery procedures, specifically the association of manual therapy with HILT and TECAR therapy, which are usually used in isolation in the recovery process for the therapy of muscle diseases. TECAR therapy, in particular, produces many beneficial effects on the body and muscle tissue. As a result of the probes used in the treatment, we can obtain perfect collaboration with manual therapy techniques, such as passive mobilisations, massages, or manipulation.

## 2. Materials and Methods

The current literature review was developed between December 2021 and April 2022 utilising the PubMed, Scopus, and Web of Science databases in accordance with the PRISMA criteria. The search formula had the following form:

(a) PubMed: (TECAR therapy) OR (HILT Therapy) OR (Manual Therapy) OR (Muscle disorders) AND (Treatment) AND (High-frequency) OR (Electric stimulation) AND (Laser treatment) OR (Muscle pain) AND (Back) AND (Knee) OR (Therapeutic effect) OR (Ultrasound Therapy) OR (Mobilisation) OR (Physical Rehabilitation) OR (knee joint effusion) OR (elastography) OR (Hydrotherapy) OR (Electromyographic activity) OR (Musculoskeletal pain) OR (therapeutic ultrasound) OR (Pain intensity evaluation) OR (Massage therapy) OR (Capacitive and Resistive Electric Transfer).
(b) Scopus: (Therapeutic effect) OR (Ultrasound Therapy) OR (Mobilisation) OR (Physical Rehabilitation) OR (knee joint effusion) OR (elastography) OR (Hydrotherapy) OR (Electromyographic activity) OR (Musculoskeletal pain) OR (Therapeutic ultrasound) OR (Pain intensity evaluation) OR (TECAR therapy) OR (HILT therapy) OR (Manual Therapy) OR (Muscle disorders) AND (Massage therapy) OR (Capacitive and Resistive Electric Transfer).
(c) Web of Science: (Muscle pain) AND (Back) AND (Knee) OR (Therapeutic effect) OR (Ultrasound Therapy) OR (Mobilisation) OR (Physical Rehabilitation) OR (knee joint effusion) OR (elastography) (TECAR therapy) OR (HILT Therapy) OR (therapeutic ultrasound) OR (Pain intensity evaluation) OR (Massage therapy) OR (Manual Therapy) OR (Muscle disorders) AND (Treatment) AND (High-frequency) OR (Electric stimulation) AND (Laser treatment) OR (Capacitive and Resistive Electric Transfer).

The records identified from the databases using the key phrases described above were created using the reference management program EndNote (X9.3.3), and the duplicate articles were also deleted with its assistance.

After that, depending on the design of the studies, we aimed to include all articles of the following type: systematic reviews, meta-analyses, case-control studies, cross-sectional studies, literature reviews, and case reports, and we excluded expert opinions, letters to the editor, and conference reports.

A word form was used to extract the data. From each article that was selected and included for review, we extracted the information that we thought fit, according to each sub-chapter, to elaborate the present literature review.

**Figure 1:**
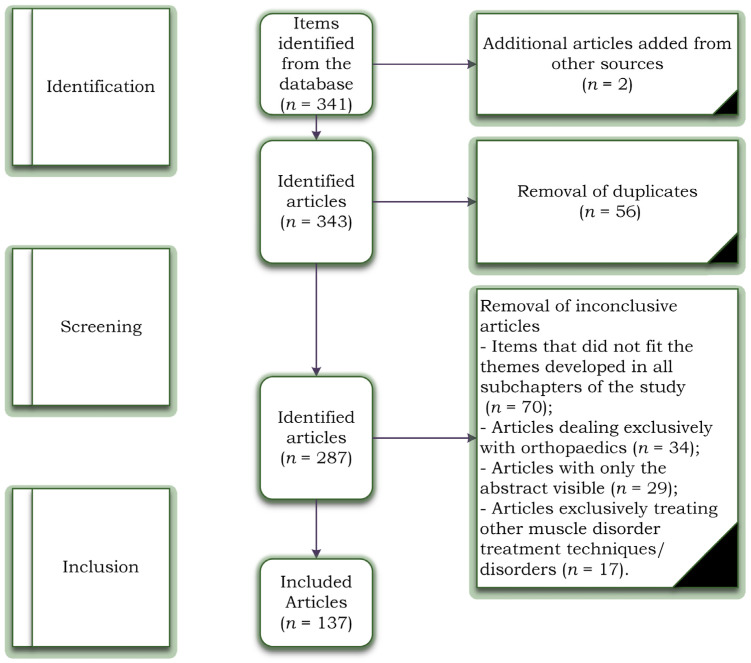
Study selection flowchart based on PRISMA criteria.

The initial search identified 341 titles in the databases described above, of which 56 duplicate articles were automatically removed, and 2 were added from other sources. The remaining 343 articles were analysed by their titles and abstracts for relevance, resulting in another 70 studies being removed. Studies were excluded at the screening stage due to items that did not fit the themes developed in all subchapters of the study (*n* = 70) because the articles dealt exclusively with orthopaedics (*n* = 34). A total of 29 articles were excluded because the articles had only the abstract visible, and 17 articles exclusively treated other muscle disorder treatment techniques/disorders. The inclusion phase resulted in 137 articles included in the study. Figure 1 represents the complete PRISMA diagram.

## 3. Manual Therapy vs. TECAR vs. HILT Therapy

Manual treatment is applied in treating muscle disorders in approximately every recovery program. Its effectiveness is debated, requiring much faster and more efficient recovery methods. Capacitive and resistive electrical transfer (TECAR), a type of diathermy, has recently been created as a form of deep thermotherapy and is used in sports medicine [21]. This device provides radio frequency energy, passing between an active and an inactive electrode and generating heat inside the body [22]. This therapy has shown that TECAR therapy is more efficient than a warm packet at increasing blood circulation, a conventional form of thermotherapy commonly used in clinical practice. Improving blood circulation plays an essential role in improving muscle recovery after fatigue. Thus, TECAR therapy can effectively improve muscle recovery after fatigue, which leads to maintaining and improving muscle flexibility [21].

Although manual therapy has been used to treat various muscle ailments since ancient times, for better and faster recovery of these ailments, more muscular stimulation is needed to have the desired effect (Figure 1). TECAR therapy combines manual therapy with deep thermotherapy, based on high-frequency electric currents, allowing for faster recovery of the affected muscles [23]. Due to the positive effects on muscle tissue, TECAR therapy seems to be a more helpful alternative in treating muscle disorders than conventional manual therapy, where cellular metabolism is not as strongly stimulated, and vasodilation is not as intense [24].

**Figure 2:**
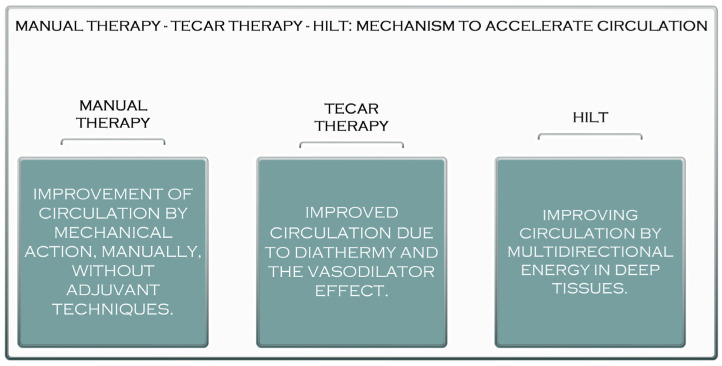
The acceleration mechanism of manual therapy, TECAR therapy, and HILT.

Laser treatment is a pain-free, non-invasive therapy that can be used to treat various clinical conditions. Laser therapy has significantly reduced acute and chronic pain, rheumatoid arthritis, chronic osteoarthritis, carpal tunnel syndrome, fibromyalgia, knee injuries, shoulder pain, and postoperative pain [25,26]. The reduction in pain after laser treatment results from its anti-inflammatory effects, the increased microcirculation and stimulation of immune processes, nerve regeneration, and the secretion of β-endorphins [26]. These properties of high-frequency laser therapy can influence the healing and regeneration of muscle tissue, which is recommended in most muscle conditions, both in athletes and non-athletes. Compared to TECAR therapy, HILT requires a much shorter treatment period, but its use with manual therapy does not seem as effective as using manual therapy with TECAR therapy because, as mentioned, the duration of treatment is short. The device does not allow a massage to be performed on the treated area. However, manipulations, massages, active or passive mobilisations, or other manual therapy techniques may be performed before or after HILT treatment (Figure 2).

## 4. The Benefits of Manual Therapy in the Treatment of Muscle Diseases

**Figure 3:**
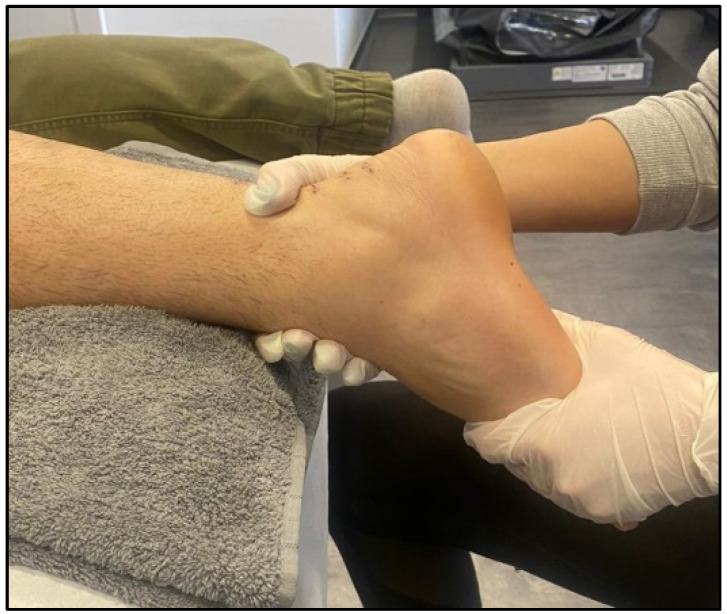
Manual therapy after Achilles tendinopathy.

Manual therapy is a technique used in physical therapy that involves using the hands to apply a force for therapeutic purposes. Manual therapy includes a wide range of therapeutic procedures such as massages, joint mobilisations/manipulations, myofascial releases, nerve manipulations, counter-stress, and acupressure (Figure 3).

Although manual therapy can be considered today as an old technique used by physical therapists, it remains one of the basic techniques of recovery used by physiotherapists in most recovery programs [27,28,29].

**Figure 4:**
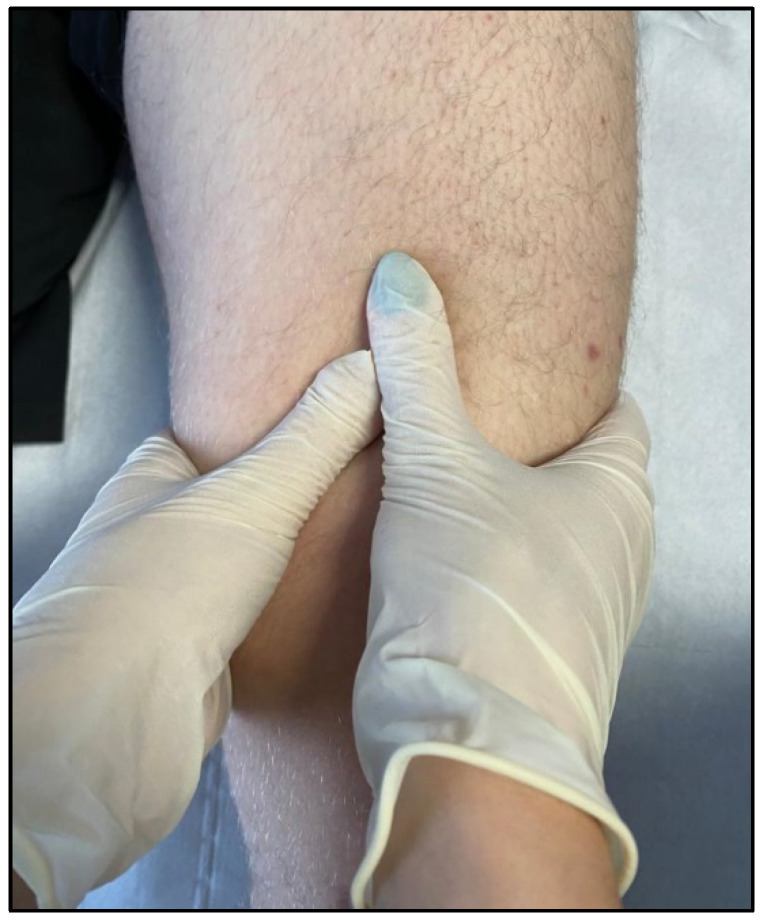
Relaxing massage of the thigh muscles (quadriceps).

Manual therapy has been used to treat individuals suffering from various diseases since ancient times. The use of hands for healing dates back to the Old Testament and appears to have been advocated in the fifth century B.C. Hippocrates suggested the application of manual therapy, including traction prone to associated manipulation of the spine. He proved the efficiency of several manual approaches and proposed modifications to force delivery characteristics such as direction, pace, and frequency [30] (Figure 4).

Currently, manual treatment is applied in treating patients with various conditions, including musculoskeletal disorders, joint dysfunctions, spinal disorders, lymphedema, musculotendinous junction disorders, cystic fibrosis, and, most importantly, after immobilisation [31].

Among the essential benefits of manual therapy are modulating pain, increasing joint mobility, especially in stiff joints and increasing range of motion, accelerating tissue repair, improving tissue stability and extensibility, reducing soft tissue inflammation, reducing muscle tension, inducing relaxation, facilitating movement, and preparing segments for exercise therapy [32]

## 5. The Benefits of HILT Therapy in the Treatment of Muscle Disorders

As previously stated, laser treatment is a non-invasive treatment with minimal risk of adverse effects [33].

High-intensity laser treatment (HILT) is primarily employed in physio-kinetic therapy therapeutic regimens [34,35,36]. The primary distinction between HILT therapy and low-intensity laser therapy is that more giant beams (power > 500 mW) are irradiated to penetrate deeper, bringing a desired large amount of multidirectional energy into the deep tissues in a short time [37,38].

Moreover, the application techniques, the treatment time, and the cost of the device are different between these two generations of laser therapy [39,40]. However, High-Intensity Laser Therapy seems to have multiple benefits in the therapy of musculoskeletal disorders, precisely due to the effects produced in the tissues and also due to the reduction in recovery time [41,42,43].

HILT provides high tissue energy, and its optical energy forms dynamic vibrations. It then generates photochemical effects, such as increased mitochondrial oxidation, and it facilitates the formation of adenosine triphosphate (ATP) and ultimately leads to the rapid absorption of oedema and the elimination of exudates by increasing metabolism and blood circulation [44,45].

HILT has its own photomechanical, photothermal, and photochemical properties and has many therapeutic effects, including oedema reduction, analgesic effects, and physical stimulation [46]. Another benefit of HILT is its greater penetration strength and depth into deep tissues [47].

High-intensity laser treatment (HILT) has lately been applied in fundamental research and clinical rehabilitation practice with promising results (Watt-level performance) (high-intensity laser, Class IV laser) [48,49].

The high-intensity laser is usually used in two ways—pulsed and continuous [50]. Each mode impacts the tissue differently and triggers different therapeutic effects. The general therapeutic effects are bio-stimulation, pain relief, anti-inflammatory effects, superficial thermal effects, and muscle relaxation [51].

The high-intensity laser delivers energy to the cells, stimulates cellular metabolism, and promotes quicker resorption of the pro-inflammatory mediators [52].

Decreasing the concentration of pro-inflammatory mediators improves capillary permeability, resulting in the total eradication of inflammation and a speedier return to daily activities and sports. The high-intensity laser can provide extremely brief heartbeats at a maximum repetition rate [53]. This property can create real pressure. Pressure waves are transported through the tissue, stimulating the free nerve endings. According to the pain control mechanism, the mechanical stimulation of free nerve terminals causes their inhibition and, as a result, pain reduction [54]. The high-intensity laser has an immediate and long-lasting analgesic effect. The energy transferred by the continuous emission of HILT to the tissue causes superficial hyperthermia and vasodilation in the treated area [55]. As the blood perfusion increases, more blood passes through the treated area, and the muscles relax. In painful indications related to muscles, such as muscle injuries and muscle contracture, the patient feels immediate relief from discomfort triggered by muscle tension, and an amplitude of movement increases immediately [56].

Bio-stimulation means stimulating the body to help enhance healing and recovery at the cellular level. Mitochondria in cells metabolise oxygen. A cascade of respiratory enzymes processes oxygen and delivers it to ATP synthase, which synthesises the body’s energy source—ATP. As a result of the quicker exchange of oxygen and metabolites caused by laser irradiation, more oxygen atoms reach the mitochondria. Mitochondria are further stimulated to synthesise ATP faster. ATP allows faster R.N.A. and D.N.A. synthesis and leads to faster recovery, faster healing, and reduced oedema in the treated area [57].

Class IV laser therapy (High-Intensity Laser Therapy) can be used to treat a range of ailments, including knee, hip, and ankle osteoarthritis; rheumatoid arthritis; shoulder pain impact syndromes [58]; hip or shoulder bursitis; lumbar disc degeneration; herniated disc sciatica; tendonitis; lateral and medial epicondylitis; plantar fasciitis; and a variety of muscle disorders [59]. Compared to other procedures used in medical recovery, high-frequency laser therapy has a limited range of contraindications, but they must be considered before starting therapy using this type of laser [60].

Pregnancy is one of the absolute contraindications of HILT therapy, and although, to date, there are no documents indicating that laser therapy is harmful to a pregnant woman or her child, to be safe and to prevent any problems, the use of this procedure in pregnancy is not recommended [61].

Another contraindication to HILT is cancer. Therefore, the use of laser therapy in a patient who has cancer or is suspected of having cancer is not recommended.

There are several relative contraindications, which each physiotherapist takes into account in the use of HILT therapy, including:

- Thyroid disorders: The thyroid is known to be sensitive to light, and although it has not yet been found to harm the thyroid gland, caution and careful dosing are recommended when using the laser [62].
- Clotting problems: Laser therapy affects blood clotting, so its use for patients with such problems should be consulted with a specialist or even replaced with another procedure [63].
- Children: Although there is no contraindication to the use of the laser for children, its dose should be adjusted according to weight [64].

## 6. The Benefits of TECAR Therapy in the Treatment of Muscle Diseases

TECAR treatment is a type of non-invasive electrothermal treatment classified as deep thermotherapy, based on the planning of electric tides in the field of radiofrequency, constituting a monopolar capacitive resistive radiofrequency of 448 kHz [65,66,67,68].

TECAR or capacitive-resistive electric transfer (CRet) is often used in the treatment of muscle, joint, and tendon injuries in traumatology and sports [67,69,70]. CRet is a non-invasive electrothermal treatment characterised as deep thermotherapy based on the application of electric flows in the radiofrequency range of 300 kHz to 1.2 MHz [71,72,73]. Unlike superficial thermotherapy, which has a relatively limited ability to penetrate muscle tissue [74,75], CRet may create heat in deep muscle tissue, hence increasing haemoglobin saturation [76]. Applying an electromagnetic field of around 0.5 MHz to the human body is responsible for this form of therapy’s physiological effects. It has been discovered that an increase in blood perfusion is associated with a rise in body temperature, and other effects, such as cell proliferation, seem to be primarily connected to the current flow [77,78]. It has been demonstrated that cell growth begins at 0.00005 A per square millimetre [69,78].

TECAR therapy is characterised by its quick action and is employed in high-performance sports because it speeds up the recovery process [79,80]. Thermal changes in the neuromotor arrangement caused by TECAR treatment generate vasodilation, minimise muscle spasms, speed up cellular metabolism, and increase the extensibility of soft tissues [81,82].

TECAR therapy is available in capacitive (C.A.P.) and resistive (R.E.S.) modes. These modes are often given with a number of immaculate medical steel probes (electrodes) that have been manufactured [83]. According to TECAR, depending on the strength of the treated tissue, the two treatment techniques generate various tissue responses. When a cushioning ceramic coating functions as a dielectric mechanism (C.A.P.) on the active electrode, energy transfer generates only heat to the superficial layers of tissue, with selective action on soft tissues with low impedance (rich in water), e.g., adipose matter, muscles, and the lymphatic system [79].

**Figure 5:**
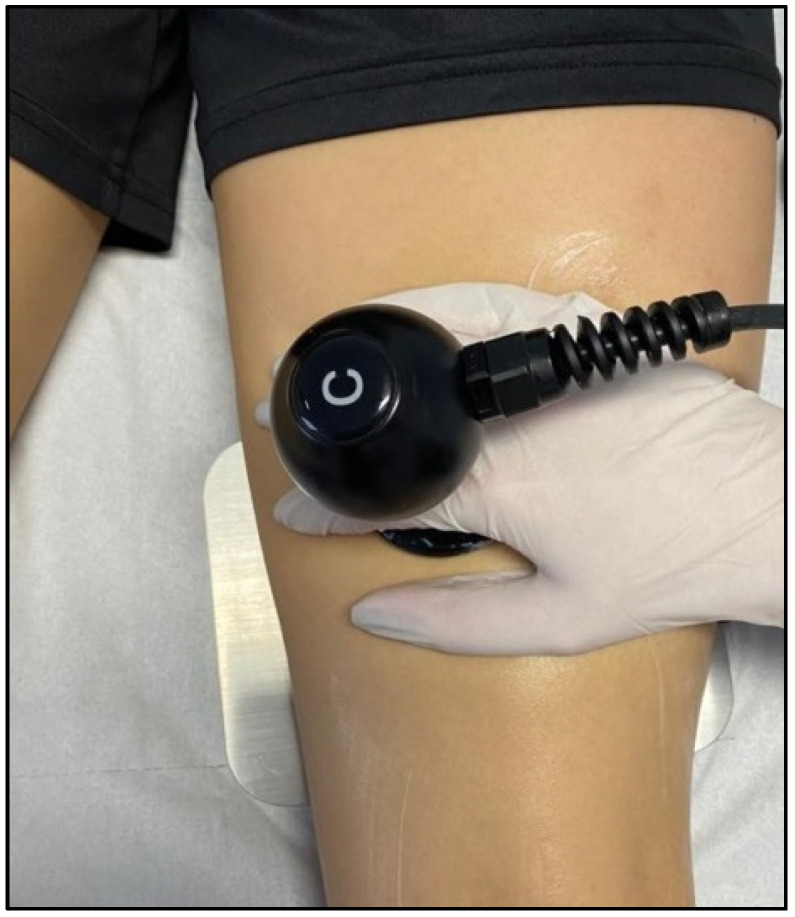
TECAR therapy in the treatment of hamstring muscles.

The resistive mode targets denser tissues with more fat and fibre (such as bones, ligaments, and tendons). High-frequency waves penetrate deep into tissues and cause a rise in exchange and temperature, and recent studies have shown the effects of radiofrequency on skin microcirculation and intramuscular blood flow [84,85] (Figure 5).

It should be noted that this new technology is a valuable tool in the therapy of different pathologies compared to other therapies used in recovery in terms of the presence and/or absence of specific positive effects while having distinctive features compared to conventional treatments [86]. It is guaranteed that TECAR therapy provides a balance between the therapist’s manual capacity and the unique energy that this technology emanates from the tissues, thus giving therapists and patients much more satisfactory results [87]. Among its positive effects is better blood circulation, which removes inflammatory catabolites [88]. TECAR therapy also substantially improves blood circulation in the peritendinous region and increases haemoglobin saturation [89]. In addition, this therapy warms deep tissues, stimulates vascularity, promotes the repose of tissues (particularly muscle tissue), and stimulates drainage (oedema and hematoma) [90]. Based on these properties, TECAR treatment is employed in most orthopaedic pathologies before the start of rehabilitation exercises [91].

Among the physiological effects of TECAR therapy are increased extensibility of collagen tissue due to reduced viscosity; reduced discomfort because of anti-irritant behaviour or release of endorphins; reduced spasms and muscle contractions due to reduced activity of secondary efferents; faster dissociation of oxygen because of more available haemoglobin [92], accompanied by a reduction in the activation energy of important chemical and metabolic reactions; vasodilation with increased local blood flow, contributing to the replenishment of oxygen and nutrients; the elimination of catabolites; and accelerated reabsorption of hemorrhagic masses [93].

## 7. Manual Therapy–TECAR–HILT Therapy: Recommendations in the Treatment of Muscle Diseases

Capacitive-resistive electrical transfer therapy (TECAR) is mainly used to treat musculoskeletal injuries [93]. TECAR is a deep thermotherapy non-invasive electrothermal therapy. It uses electric currents at radio frequencies ranging from 300 kHz to 1.2 MHz [90]. This current can induce warming of deep muscle tissue, improving haemoglobin saturation, increasing deep and superficial blood flow, vasodilation, increased temperature, fluid removal, and cell proliferation [94]. Responses such as increased blood perfusion appear to be associated with increased temperature, which is generated due to a physical reaction generated by current flow (Joule effect) [95]. Increased cell escalation, nevertheless, seems to be associated with the current flow rather than increased temperature. Precisely due to these effects produced by TECAR therapy, it seems ideal for treating all muscle disorders compared to other therapies used, especially in athletes [96].

As mentioned above, TECAR therapy offers two different modes of treatment: capacitive and resistive. Different therapy methods trigger various tissue reactions relying on the treated tissue’s strength [92]. A ceramic layer insulates the capacitive mode, and energy transfer creates heat in the surface tissue layer’s selective action in the tissues with low impedance (rich in water). The resistive mode does not have an insulating ceramic layer; the radio frequency energy travels immediately across the physique in the orientation of the unemployed electrode, causing heat to be generated in the more profound and more resilient tissues (through lower water content) [97,98]. Given the benefits induced in part by each electrode (capacitive and resistive), the capacitive mode seems ideal in the therapy of muscle injuries, muscle cramps, spasms, contractions, and myalgias, as well as for reducing post-exercise muscle tension.

Unlike surface thermotherapy, which has a minimal ability to reach muscle tissue, TECAR therapy can generate heat in deep muscle tissue, improving haemoglobin saturation [99]. The physiological effects of this sort of therapy are caused by exposing the human body to an electromagnetic field of around 0.5 MHz. This approach has improved blood circulation, deep and surface temperatures, vasodilation, lymphatic effects, and increased cell proliferation [100]. An increase in blood strain has been noticed and is related to rising temperatures, but other effects—such as cell proliferation—seem to be mainly related to the stream of the current [101].

The mechanism of action of HILT Is not precise. It is considered to have both photochemical and photothermal effects, resulting in an anti-inflammatory, anti-edematous, analgesic, and repairing treatment. It is suspected that the analgesic influences of HILT are based on various mechanisms of action, such as slowing down the transmission of pain stimuli and increasing the production of morphine-mimetic substances in the body [102].

This treatment provides changes in blood flow and an expansion in the permeability of blood vessels and accelerates the response of cellular metabolism [103]. Furthermore, the photochemical consequences of HILT can promote collagen formation in the structure of tendons as well as enhance bloodstream and vascular resistance permeability, causing anti-inflammatory effects [104]. Thus, HILT can support the maintenance of the destroyed tissue and eliminate painful stimuli. Regarding muscle disorders, HILT is recommended in their treatment precisely because of the effects produced, initially to relieve pain and accelerate the healing process of affected muscle tissue [105].

**Figure 6:**
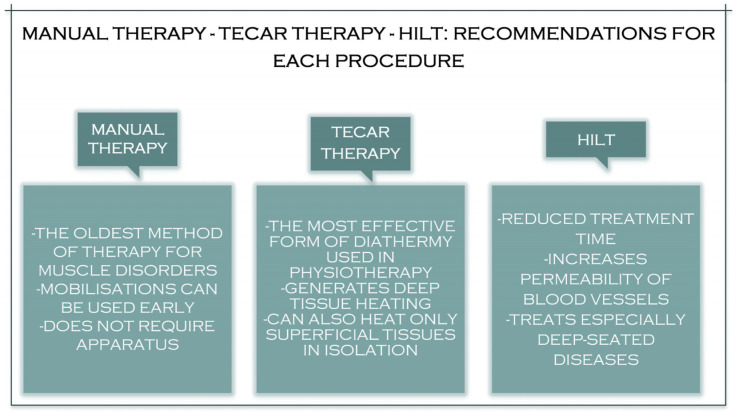
The advantages of recommending manual therapy, TECAR, and HILT therapy.

Manual therapy appears to have been utilised in the treatment of muscle ailments since ancient times, with physio-kinetic therapists using it from the acute stages of the disease before thermotherapy and electrotherapy became widespread [106]. The manipulation of muscle tissue primarily helps to increase elasticity and prevent muscle atrophy and joint stiffness caused by trauma [107,108]. However, massages, especially in the severe phase of muscle injuries, are recommended and have many beneficial effects in the therapy of muscle contractures [109]. Passive, active, passive–active, and active–passive mobilisations are among the most used manual therapy techniques in the therapy of muscle diseases, being included in the protocol for the use of muscle diseases [110]. These techniques must be launched as quickly as necessary because, with their help, we can prevent the installation of joint stiffness and deficits [111] (Figure 6).

## 8. Manual Therapy–TECAR–HILT Therapy: Methods of Use in the Treatment of Muscle Diseases

Skeletal muscles allow people to move and perform daily activities. They play a crucial role in respiratory mechanics and help maintain posture and balance. They also protect vital organs in the body [112].

Various medical conditions result from abnormal skeletal muscle function [113]; these conditions include myopathies, paralysis, myasthenia gravis, urinary and/or intestinal incontinence, ataxia, weakness, tremors, and other disorders [114]. Nerve diseases can cause neuropathy and skeletal muscle dysfunction. Furthermore, skeletal muscle/tendon ruptures can occur acutely in high-level athletes or recreational sport participants and can cause a significant disability in all patients, regardless of activity status [115,116].

**Figure 7:**
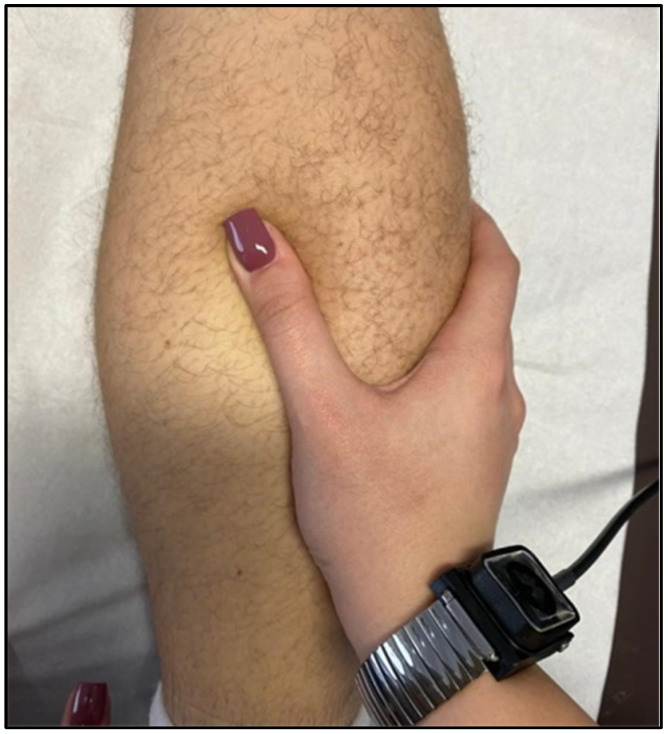
TECAR therapy with massaging calf muscles.

TECAR therapy is one of the most beneficial treatments used in muscle diseases due to the multitude of positive effects it brings to muscle tissue [117] (Figure 7).

Moreover, TECAR therapy has several uses for the better healing of muscle tissue. One of these is the one used by TECAR Winback therapy, where the positive electrode consists of a bracelet that the therapist places around his or her radio-carpal joint, using his or her hand to perform the treatment. This function offers the therapist better manipulation of the muscle tissue, working passively with the patient to mobilise the joint and simultaneously combining manual therapy with TECAR therapy. TECAR therapy also has fixed applicators, which are fixed in the patient in the proximity of the disease, thus offering the therapist the possibility of performing a complex treatment, combining the advantageous influences of diathermy with manual therapy and passive therapy mobilisations.

HILT (High-Intensity Laser Therapy) is a convenient, non-invasive, and painless way that improves joint mobility [118], stimulates adequate tissue deepening [119], and offers anti-inflammatory, analgesic, and other valuable benefits in tissue healing [120]. In addition, the anti-inflammatory, anti-edematous, and analgesic effects are practical in the first treatment sessions [121]. Treatment sessions are short and recommended to be conducted daily for a faster effect. Depending on the brand of laser used, HILT can be used both in direct contact with the skin and at a distance of a few millimetres from it. The patient feels a warm sensation in the treated region, which is easily tolerated.

Another benefit of HILT, for which its use is recommended, is given by the depth at which the laser penetrates the tissue, having the ability to penetrate superficial tissues, reaching the site of the lesion to be treated [122,123,124].

HILT is a therapy used in isolation and in combination with other thermotherapy and electrotherapy procedures [125,126]. Moreover, in most cases, it is used in conjunction with physical therapy, playing an essential role in modulating pain [127].

Manual therapy is used in all muscle diseases, isolation, and other procedures, especially in physiotherapy [128,129]. Its method of use varies, and the physiotherapist can use his or her own hands to treat various devices/accessories specific to physical therapy. Massaging is very effective in muscle contractions, fatigue, or muscle loads [130] combined with cryotherapy. Physiotherapists also use joint mobilisation during physical therapy sessions to maintain joint mobility and to make the patient aware of the movement, especially after surgery [131].

Manual resistance physical exercises performed by the physiotherapist are also practical, especially in the early stages of recovery from muscle disorders, before the patient can perform exercises with elastic or mechanical resistance.

## 9. Manual Therapy–TECAR–HILT Therapy: Contraindications

Although TECAR therapy currently seems to be one of the most beneficial therapies used in recovery [132], it also has several contraindications that the therapist must consider before starting treatment with this type of therapy [133]. Among the most critical absolute contraindications of TECAR therapy are patients with pacemakers, patients with hemorrhagic gastrointestinal ulcers, patients with infusion pumps and electric cable implants, patients in the first six months of pregnancy, in the treatment of localised cancerous area/tumours, patients with allergic reactions to certain substances in the conductive cream, patients with deep-vein thrombosis, patients with uncontrolled ischemic heart disease, patients with local pulmonary embolism, patients with phlebitis, and for treating areas with bleeding or where the skin has partial or open wounds (Figure 7).

In addition to the contraindications of TECAR therapy, there are several precautions that the therapist must take into account when using this type of therapy. One of them is removing metallic materials from the patient, such as bracelets, earrings, and watches, especially from the area where the treatment is to be performed. It is also good to know that TECAR therapy is recommended for adults, as it can have adverse effects on growth cartilage, although this has not yet been demonstrated [134].

**Figure 8:**
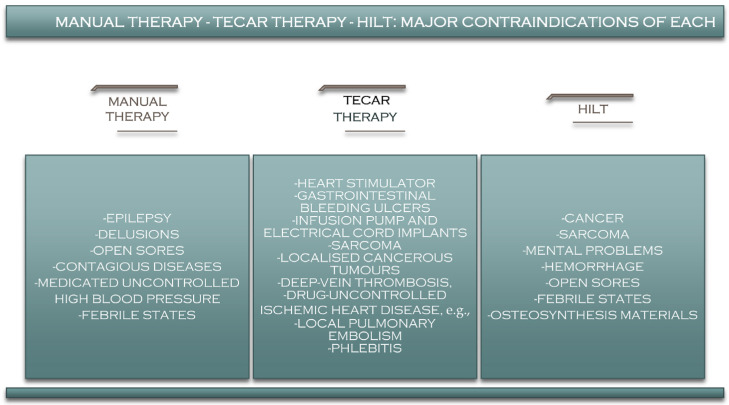
Contraindications of manual therapy, TECAR, and HILT therapy.

HILT also has several significant contraindications that physicians and physiotherapists should consider before recommending or performing a high-intensity laser treatment. As with TECAR therapy, pregnancy is a contraindication to high-intensity laser treatment, and although there is no evidence that laser therapy can harm the pregnant woman or the child, for safety reasons, this type of treatment is recommended in the case of pregnancy [135]. Cancer is also a contraindication in the case of HILT therapy, as it is in the case of other therapies, because the effects it can have on cancer cells are not known [136]. In some countries, however, such as France, the laser is used to treat mucosal pain (Figure 8).

Unlike other therapies, HILT is also recommended for patients with pacemakers, implants, screws and plaques and even for children [137].

Manual therapy a procedure with some of the fewest contraindications, and it can be used in almost any category of patient (Figure 8). However, it is recommended to avoid using it in patients with uncontrolled hypertension, mental problems, delirium, or epilepsy.

## 10. Limitations of the Study

This study is a description of the theoretical framework of how these techniques work rather than what has been demonstrated by research studies regarding valid research designs and reliable and valid outcome measures. Our research includes studies with various pathologies with which these therapeutic modalities are used, without going into the details of their effectiveness.

## 11. Conclusions

Given the many benefits that capacitive and resistive electrical transfer therapy has on muscle disorders, it should be integrated into most recovery protocols to enhance the healing process and to make rehabilitation faster and more straightforward.

However, manual therapy remains as one of the safest and most frequently used methods by physical therapists, with very few contraindications and allowing the therapist to feel and treat with his or her own hands. If combined, these therapies can have much better results, such as the effects of manual therapy (reducing muscle tension, inducing relaxation, and increasing mobility through passive movements performed by the physical therapist) along with the many effects resulting from TECAR muscle tissue therapy (increasing cellular metabolic processes, activating the body’s natural repair processes, improving blood flow and improving pain), and also with the effects produced by HILT (accelerating cellular metabolism, faster resorption of pro-inflammatory mediators). HILT is also beneficial in treating muscle diseases, affecting blood circulation and cellular metabolism, thus accelerating tissue regeneration.

TECAR therapy combined with manual therapy in the management of muscle disorders provides excellent coordination in the healing process, which is beneficial for all types of muscle diseases. Through the variety of benefits of each one, more positive results can be obtained, unless used specifically with only one of the two therapies. Moreover, manual therapy combined with HILT appears to have similar effects to the previous combination but is not as pleasant for the patient. TECAR therapy with HILT and manual therapy may be ideal for treating muscle disorders to accelerate healing, maintain tissue elasticity and joint mobility, and restore the patient to pre-injury fitness.

## Data Availability

Not applicable.
